# COM2/351: Multimedica - The German Online Information Provider for Physicians: 3 years of experience and perspectives for the future

**DOI:** 10.2196/jmir.1.suppl1.e15

**Published:** 1999-09-19

**Authors:** L Schaeffer, K Bob

**Affiliations:** ^1^HOS Multimedica, BerlinGermany

**Keywords:** Online Medical Information System, Virtual Private Network, Portal Site for Physicians, Online Publishing, Multimedica

## Abstract

The exponential growth of medical information together with the high turn over of medical knowledge creates an updating pressure that print media are no longer able to provide. Corresponding to the physicians needs for rapid available information and high quality of information standards, the aim of a professional online service must be to supply its defined target group with international, up-to-date, reliable and well-structured information. Multimedica is a virtual private network for physicians and pharmacists (e.g. an Internet-based password-protected medical online service) in the German language. With over 150,000 pages covering 35 speciality fields (e.g. scientific journals in full text, full text online versions of numerous textbooks, address and drug data bases etc), Multimedica is able to react to the fast growing need for information, education and communication in medicine. The high quality standard is guaranteed by bringing together medical information providers, such as scientific publishers, medical associations and renowned specialists. The availability of online teleconsultation covering more than 60 speciality fields provides the possibility of information exchange and gives an uncomplicated way for general practitioners to get a second opinion, e.g. an expert advice not depending on consulting hours or availability of the specialist on the telephone. The ultimate aim of this project is to establish Multimedica as an integral part of the doctors' decision-making process, as well as providing a comfortable tool to ensure CME (Continuous Medical Education) for every physician. Up to now more than 20,000 physicians are using the service. In this paper we will discuss in detail the background, aims and objectives, needed resources, as well as our first 3 years of experience.

